# In-utero transmission of SARS CoV-2 infection during delta variant outbreak: first Australian experience

**DOI:** 10.1007/s15010-022-01764-4

**Published:** 2022-02-08

**Authors:** Robert C. Duguid, Srinivas Bolisetty, Fatima Anne Perez, Tim Schindler, Pamela Palasanthiran, Zin Naing

**Affiliations:** 1 Newborn Care Services, Royal Hospital for Women, Sydney, Australia; 2 Department of Immunology and Infectious Diseases, Sydney Children’s Hospital, Sydney, Australia; 3 Serology and Virology Division (SAViD), NSW Health Pathology Randwick, Prince of Wales Hospital, Sydney, NSW 2031 Australia

Dear Editor,

We read the case report published by Kularni et al. [1] in *Infection* with great interest and respond to it with a case of our own that provides further evidence of the potential for in-utero transmission of SARS-CoV-2. Our case occurred during the current outbreak of the Delta variant in Sydney, Australia. To our knowledge this report also describes the first case of neonatal COVID in an Australian Tertiary Newborn Care Centre.

The mother is a 25-year-old woman who has no significant past medical history. The current pregnancy was uncomplicated prior to SARS-CoV-2 infection. Her 29-year-old husband first tested positive for SARS-CoV-2 after developing mild symptoms of COVID-19. Three days later, the mother developed a sore throat and nausea and subsequently tested positive for SARS-CoV-2. Both parents were unvaccinated and had mild COVID-19 illness that did not require hospitalisation. On day 8 of COVID-19 illness, the mother presented to the hospital with abdominal pain, vaginal bleeding and decreased foetal movements. Placental abruption was presumed and due to an abnormal cardiotocography (CTG), the baby was delivered by pre-labour caesarean section at 32 weeks and 6 days gestation with a birthweight of 1933 g (73rd percentile). The female infant developed respiratory distress soon after birth requiring bubble continuous positive airway pressure (CPAP) therapy followed by intubation, mechanical ventilation and surfactant therapy. She was extubated to CPAP at 24 h of age and subsequently weaned to air on day 5 of life. She continued to have intermittent rhinorrhoea and occasional diarrhea up to day 15 of life. The remainder of the course was consistent with prematurity.

**Fig. 1:**
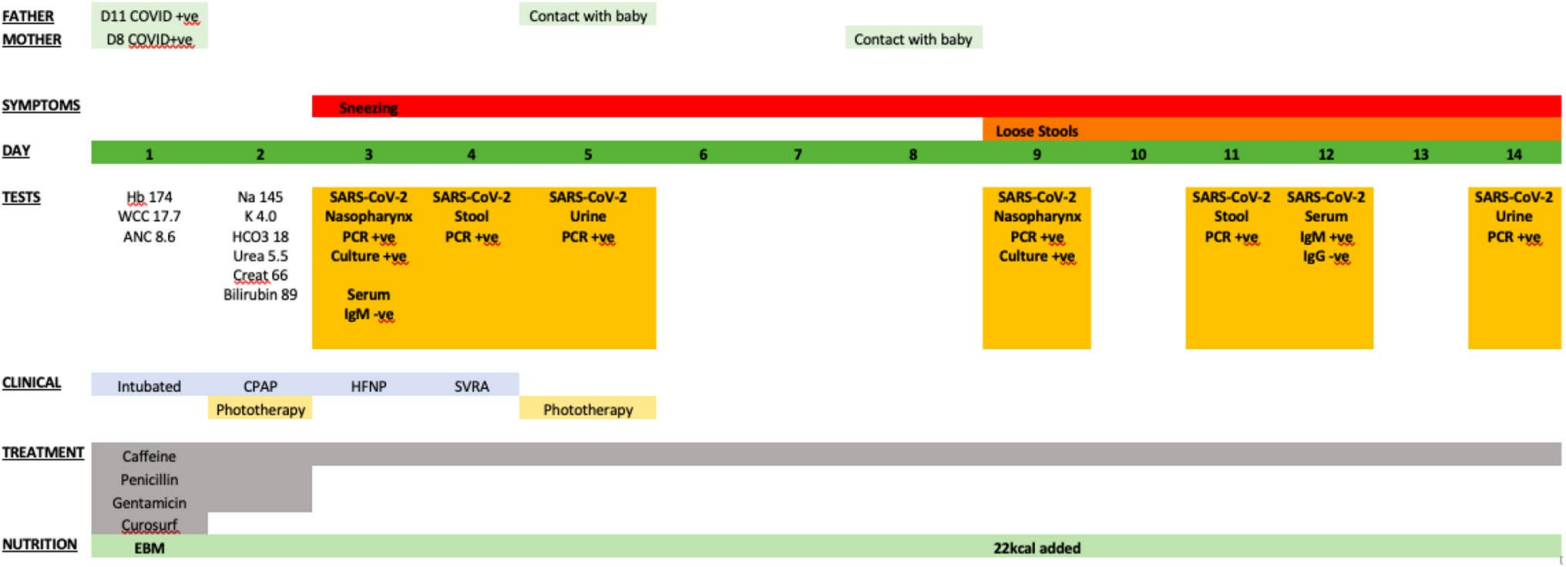
Timeline of symptoms and laboratory parameters of the SARS-CoV-2 infected newborn. *Hb* haemoglobin (g/L), *WCC* white blood cell count (× 10^9/L), *ANC* absolute neutrophil count (× 10^9/L), *EBM* expressed breast milk, *Na* sodium (mmol/L), *K* potassium (mmol/L), *HCO3*  bicarbonate (mmol/L), *Urea* urea (mmol/L), *Creat* creatinine (µmol/L), *Bilirubin* bilirubin (µmol/L), *CPAP* continuous positive airway pressure, *PCR* polymerase chain reaction, *HFNP* high flow nasal prongs, *SVRA* spontaneous ventilation in room air), 22 kcal = human milk fortifier, 22 kilocalories per ounce of expressed breast milk (EBM)

National COVID-19 guidance for neonatal units in Australia recommend neonates of COVID-19 positive mothers are not routinely tested, but if clinically indicated (as in our case), the case is discussed with the local infectious diseases team. This recommendation was made based on concern that day 0 testing may represent maternal contamination and not infection. There is no clear guidance or recommendation for testing of amniotic fluid, placenta or cord blood. The infant was first tested for SARS-CoV-2 RNA by real-time PCR (Seegene Inc., Seoul, Korea) of a nasopharyngeal sample at 60 h of life and this was positive. The specimen demonstrated a low cycle threshold (Ct = 14 for E-, N-, and RdRP/S-gene) indicating a high viral load. SARS-CoV-2 RNA was also detected in faecal and urine samples, with Ct values ranging from 24 to 34. Another nasopharyngeal sample collected at 9 days of life had SARS-CoV-2 RNA detected at Ct = 18 for ORF1a and E genes (Roche Diagnostics, Indianapolis, USA), indicating the viral load remained high. On day 12 of life Abbott Architect SARS-CoV-2 (anti-spike) IgM EIA (Abbott Laboratories, Chicago, USA) was positive and Euroimmun anti-SARS-CoV-2 (anti-spike) IgG EIA (Euroimmun, LüBeck, Germany) was negative. The infant then tested positive for Abbott Architect SARS-CoV-2 IgG EIA (Abbott Laboratories, Chicago, USA) on day 28 of life, suggestive of either seroconversion or transfer of maternal antibody via breastmilk. The clinical history and investigations are summarised in Fig. 1.

Potential mechanisms of maternal transfer of SARS CoV-2 to the infant include (1) in-utero transmission either transplacentally or via ingestion by the fetus of viral particles in amniotic fluid, (2) intrapartum through direct contamination of infected amniotic fluid or maternal secretions and (3) post-partum through direct contact^2^. Reported rates of vertical transmission are 2% [2], however, these data are prior to the emergence of the delta variant. In our case, the mother gave birth on day 8 of illness, likely after her period of peak infectiousness. Evidence suggests that viral load peaks within the first 2 days after symptom onset [3]. This likely occurs even earlier with the Delta variant [4]. Due to this, we hypothesise this case represents an example of in-utero transmission. Pathophysiological plausibility and multiple case reports of probable in-utero transmission have been recently well summarised [5]. The cell-membrane associated angiotensin-converting enzyme 2 (ACE-2) receptor and transmembrane protease serine 2 (TMPRSS2) required for SARS-CoV-2 cellular entry have been identified in placental cells [6]. Vascular damage to the placenta could also allow SARS-CoV-2 to reach the fetus without placental cell infection via amniotic fluid.

The low cycle threshold (Ct = 14) of our case’s sample on day 3 of life and isolation in viral culture suggests a high viral load and recent infection rather than transient contamination. Neonatal infection was confirmed with persistence of SARS-CoV-2 isolated in viral culture from a nasopharyngeal sample on day 9 of life. Viral culture was performed in a certified PC3 certified laboratory in Sydney, Australia using the methods described in Stelzer-Braid et al. [7]. A modified cell line, HEK-293 T cells transduced with ACE2 and TMPRSS receptors, was maintained in Dulbecco’s Modified Eagle Medium (DMEM) (Life Technologies) supplemented with 10% FBS and 1 × PSG. On the day of virus culture, cell suspension was prepared in DMEM (Life Technologies) supplemented with 2% FBS and 1 × PSG. 10^4^ cells from cell suspension were dispensed into each well of a 96-well flat-bottom tissue culture plate. HEK-293 T cells were then inoculated with 50µL clinical sample (filtered through 0.22 µm filter at 10,000 g for 5 min) and incubated at 37 °C with 5% CO_2_. Signs of cytopathogenic effect (CPE) were monitored at day 1 and day 4 post-infection. CPE was observed from day 1 of culture on the nasopharyngeal sample taken from our case on day 3 of life. Whole genome sequencing (Oxford Nanopore Technologies) of the day 3 clinical sample confirmed that the case was infected with the Delta variant (pangolin lenage:AY.39.1; Scorpio call: Delta- B1.617.2-like).

Postnatal transmission is the most commonly described mode of transmission in the literature [2]. We deem this unlikely in our case, as the mother and child were separated immediately after birth and did not have any close contact until after the mother was de-isolated on day 14 of COVID-19 illness. The father was not present at the delivery and did not have any contact with the baby until day 5 of life. The baby received EBM prior to testing positive for SARS-CoV-2 on day 3 of life, however, replication-competent virus in breast milk has not been described in the literature [8]. Hospital acquired infection inside our Newborn Care Centre is unlikely, because all staff members involved in the care of the baby were tested negative to SARS-CoV-2 as part of our surveillance testing program, and all were fully vaccinated. Intrapartum transmission is also less likely in the context of a pre-labour caesarean section in our case.

Ultimately, we cannot prove or disprove in-utero foetal exposure as we did not perform any testing in-utero or before 24 h of age. Unlike in the case described by Kulkarni et al. [1] we did not test placental or cord blood samples which would strengthen the case for in-utero transmission. Placental abruption makes peri-partum infection possible. However, due to the timing of peak infectiousness of the Delta variant and the isolation of the case from the parents at delivery, we feel in-utero infection is the most likely mode of transmission in our case. Despite previously reported low rates vertical transmission of SARS-CoV-2 in the literature and in-particular scepticism about the potential for in-utero transmission, we believe that these recent symptomatic neonatal cases in the context of the Delta variant warrant further research.
